# Assessment of Cancer Survivors’ Experiences of Using a Publicly Available Physical Activity Mobile Application

**DOI:** 10.2196/cancer.5380

**Published:** 2016-05-31

**Authors:** Patrycja Puszkiewicz, Anna L Roberts, Lee Smith, Jane Wardle, Abigail Fisher

**Affiliations:** ^1^ Health Behaviour Research Centre Department of Epidemiology & Public Health University College London London United Kingdom; ^2^ The Cambridge Centre for Sport and Exercise Sciences Department of Life Sciences Anglia Ruskin University Cambridge United Kingdom

**Keywords:** cancer survivors, mobile applications, mHealth, physical activity, sleep

## Abstract

**Background:**

Regular participation in physical activity (PA) is associated with improved physical and psychosocial outcomes in cancer survivors. However, PA levels are low during and after cancer treatment. Interventions to promote PA in this population are needed. PA mobile apps are popular and have potential to increase PA participation, but little is known about how appropriate or relevant they are for cancer survivors.

**Objective:**

This study aims to (1) assess recruitment, study uptake, and engagement for a publicly available PA mobile app (GAINFitness) intervention in cancer survivors; (2) assess cancer survivors’ attitudes towards the app; (3) understand how the app could be adapted to better meet the needs of cancer survivors; and (4) to determine the potential for change in PA participation and psychosocial outcomes over a 6-week period of using the app.

**Methods:**

The present study was a one-arm, pre-post design. Cancer survivors (N=11) aged 33 to 62 years with a mean (SD) age of 45 (9.4), and 82% (9/11) female, were recruited (via community/online convenience sampling to use the app for 6 weeks). Engagement with the app was measured using self-reported frequency and duration of usage. Qualitative semi-structured telephone interviews were conducted after the 6-week study period and were analyzed using thematic analysis. PA, well-being, fatigue, quality of life (QOL), sleep quality, and anxiety and depression were self-reported at baseline and at a 6-week follow-up using the Godin Leisure Time Exercise Questionnaire (GLTEQ), the Functional Assessment of Cancer Therapy-General (FACT-G), the Functional Assessment of Chronic Illness Therapy (FACIT)-Fatigue Scale Questionnaire, the Health and Quality of Life Outcomes (EQ5D) Questionnaire, the Pittsburgh Sleep Quality Index (PSQI), and the Hospital Anxiety and Depression Scale (HADS), respectively.

**Results:**

Of the people who responded to the study advertisement, 73% (16/22) agreed to participate and 100% (11/11) of the participants who started the study completed all baseline and follow-up outcome measures and the telephone interview. On average, participants used the app twice a week for 25 minutes per session. Four themes were identified from the qualitative interviews surrounding the suitability of the app for cancer survivors and how it could be adapted: (1) barriers to PA, (2) receiving advice about PA from reliable sources, (3) tailoring the application to one’s lifestyle, and (4) receiving social support from others. Pre-post comparison showed significant increases in strenuous PA, improvements in sleep quality, and reductions in mild PA. There were no significant changes in moderate PA or other psychosocial outcomes.

**Conclusions:**

All participants engaged with the app and qualitative interviews highlighted that the app was well-received. A generic PA mobile app could bring about positive improvements in PA participation and psychosocial outcomes among cancer survivors. However, a targeted PA app aimed specifically towards cancer survivors may increase the relevance and suitability of the app for this population.

## Introduction

It is estimated that 1 in 2 people born in the United Kingdom (UK) after 1960 will develop cancer during their lifetime [1]. However, improvements in early detection, diagnosis, and treatment mean that cancer mortality is falling, with 50% of people being diagnosed with cancer now surviving more than 10 years. The rising incidence and falling mortality of cancer has led to an increase in the number of cancer survivors. In the context of cancer, “a person is considered to be a survivor from the time of diagnosis until the end of life” [2]. In the United Kingdom, there are currently over 2 million people living with or beyond a cancer diagnosis; this has doubled in the last 40 years and continues to increase by 3% each year [3].

Fatigue [4], poor sleep quality [5], reduced quality of life (QOL) [6,7], pain [8], physical side effects (eg, lymphedema) [9,10], depression, anxiety and/or fear of cancer recurrence [11-14] are common among cancer survivors as a consequence of diagnosis, treatment, and side effects. The Living With and Beyond Cancer (LWBC) Program was formed in partnership between the UK Department of Health, the National Health Service (NHS), and a large national cancer charity, Macmillan Cancer Support, to improve the overall care and support needs of the growing population of cancer survivors. One key area of focus for the LWBC Program is to promote participation in physical activity (PA) among cancer survivors due to the accumulating body of evidence illustrating the benefits of PA for this population. Evidence includes an increased chance of survival (both cancer-specific [15-17] and all-cause survival [16,17]), reduced risk of cancer recurrence [16], improved physical and psychological health and consequences of treatment (eg, fatigue [18-22], sleep disturbance [18,23], pain [18], muscle strength [22,24-26], physical functioning [27], well-being [18], QOL [18-20,22,28,29], anxiety and depression [22,30]) and a reduced risk of comorbidities (eg, hypertension, cardiovascular disease, and diabetes) [31]. Much of this research has involved breast, prostate, and colorectal cancer survivors and so the evidence is strongest for these cancer types. Evidence of the benefits of PA for survivors of other cancer types (eg endometrial [32-36], hematological [37], and head and neck [26] cancers) is emerging.

Despite the benefits of PA, only 35% of cancer survivors engage in at least 2 hours of PA per week compared to 45% of those without a history of cancer [38]. The majority of cancer survivors do not meet the recommended minimum guideline of 150 minutes of moderate to vigorous PA per week, although this varies by cancer type [39]. Furthermore, PA levels fell and sedentary behavior rose in those who received a cancer diagnosis compared to those who did not in the English Longitudinal Study of Ageing [40]. The fall in PA participation as a result of cancer is likely due in part to the experienced deterioration in health and well-being and common side effects, such as cancer-related fatigue [41]. Fatigue is one of the most commonly reported and debilitating side effects of cancer treatment and can continue for many months or years after completion of treatment [4,42].

The majority of PA interventions involve considerable contact between health care practitioners and fitness professionals delivering the PA program to participants. They often use face-to-face delivery methods and/or frequent structured support with a health professional or member of the research team [43-47]. Many of these intervention studies demonstrate promising results in improving participation in PA and provide evidence of the associated benefits. However, such approaches also face the challenge of being resource-intensive, expensive, and limited in terms of the number of cancer survivors who are able to access them. Therefore, a low-cost and broad-reaching strategy is warranted.

The rising use of mobile phones and mobile technology has afforded the opportunity to develop a relatively low-cost approach to intervention delivery with the potential to reach a large number of users. The most recent Ofcom report (2015) [48] revealed that 66% of UK adults own a mobile phone, a 27% increase since 2012, demonstrating the rapidly rising number of mobile phone owners. The report also revealed that 49% of 55 to 64 year olds and 17% of people aged over 65 own mobile phones, a number which is accelerating rapidly and expected to continue. Many people use mobile apps to support or motivate a healthy lifestyle [49], and previous studies have found mobile health (mHealth) interventions using apps to be successful in a range of health contexts including weight loss [50] and management of diabetes [51]. PA apps are particularly popular, with an estimated 1 in 5 mobile phone users having installed at least one PA app on their mobile device [52]. Despite the vast number of PA apps available for download (eg, via the App Store or Google Play), there are very few specifically aimed at improving PA participation among cancer survivors. Cancer survivorship apps currently available to the public tend to relate to cancer-related information, accessing and storing plans for treatment and follow-up care, patient health records, symptom tracking and monitoring, and connecting cancer survivors for peer-support. There is a lack of cancer-related apps that are supported by scientific evidence [53]. While some of the survivorship apps mention PA, currently there are very few with a specific focus on improving PA participation among cancer survivors, particularly outside of the research context. In a mixed-methods study by Hong and colleagues involving 112 cancer survivors, the researchers collected data to inform the design of a website (also accessible via mobile devices) to promote PA among older cancer survivors (iCanFit) [54]. The researchers found this group welcomed the idea of using their mobile phone and the Internet to improve their PA participation, as well as to set and track their fitness goals online. A pilot of the iCanFit program with a sample of older cancer survivors (aged 60 to 78) revealed significant improvements in participants’ QOL and participation in PA [55]. These findings demonstrate the acceptability and ability of Internet- and mobile-based interventions to promote PA among older cancer survivors.

Currently there are few mobile apps specifically aimed at promoting PA among cancer survivors, particularly outside of the research context. Therefore the aims of this study are (1) to assess recruitment, study uptake, and engagement for a publicly available PA mobile app intervention amongst breast, prostate, and colorectal cancer survivors; (2) to assess the attitudes of breast, prostate, and colorectal cancer survivors towards a publicly available PA app; (3) to understand how a PA app for the general population could be adapted to better meet the needs of cancer survivors; and (4) to determine the potential for change in PA and psychosocial outcomes (fatigue, well-being, sleep quality, QOL, anxiety and depression) which could be tested in a future randomized controlled trial (RCT).

## Methods

### Study Design

This study uses a one-arm pre-post design with a 6-week follow-up using both qualitative and quantitative techniques. Techniques were selected to ascertain qualitative feedback for intervention development and to model the data collection and intervention process, and outcomes in accordance with the Medical Research Council (MRC) guidance for development of complex interventions [56].

### Participant Recruitment

Participants were recruited via posters, short recruitment messages on the Cancer Research UK forum, and social media cancer support groups using online and community-based methods. Eligibility criteria included adults aged 18 years or older who have received a diagnosis of breast, prostate, or colorectal cancer and who have finished primary curative treatment. As the mobile app was only available on the iOS operating system, participants were also required to own an iPhone to test the application during the study period. The recruitment period lasted 10 weeks.

### Mobile Application

**Figure 1:**
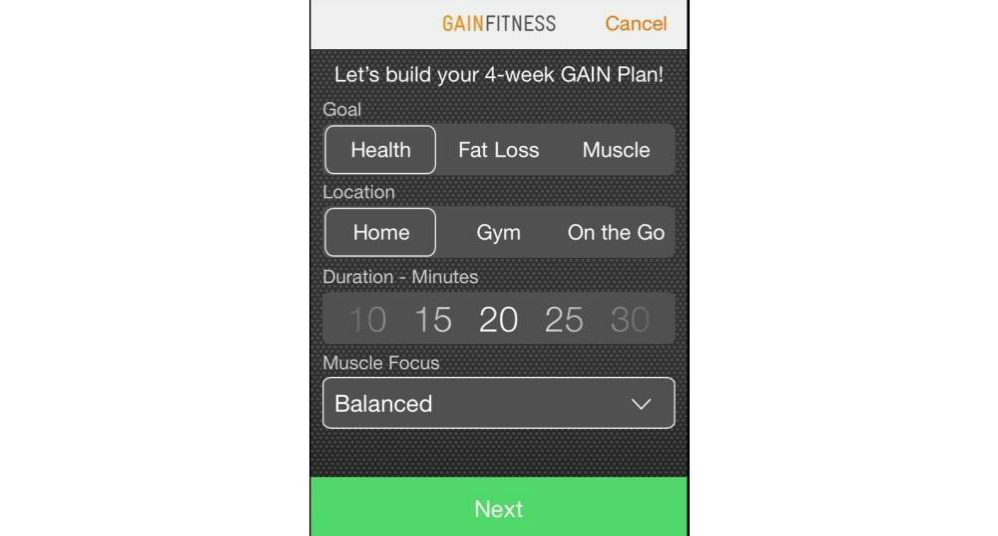
Identify exercise goal, location, duration, and muscle group.

**Figure 2:**
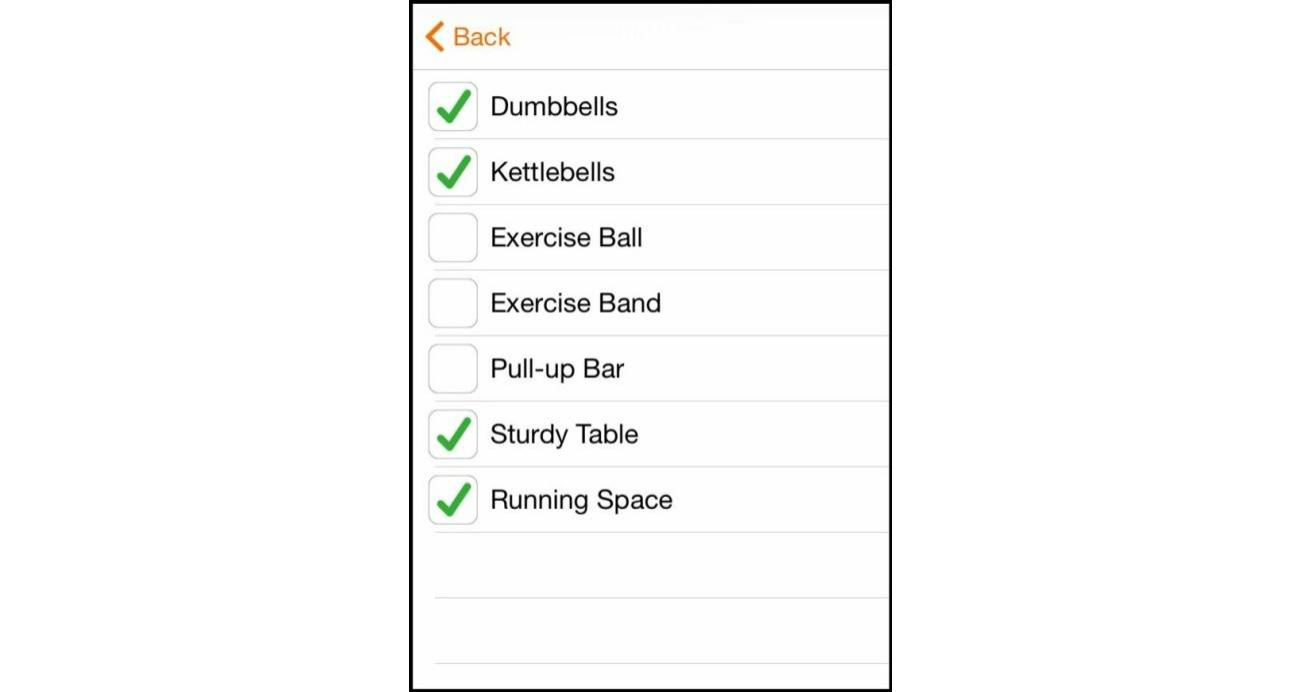
Identify accessible exercise equipment.

**Figure 3:**
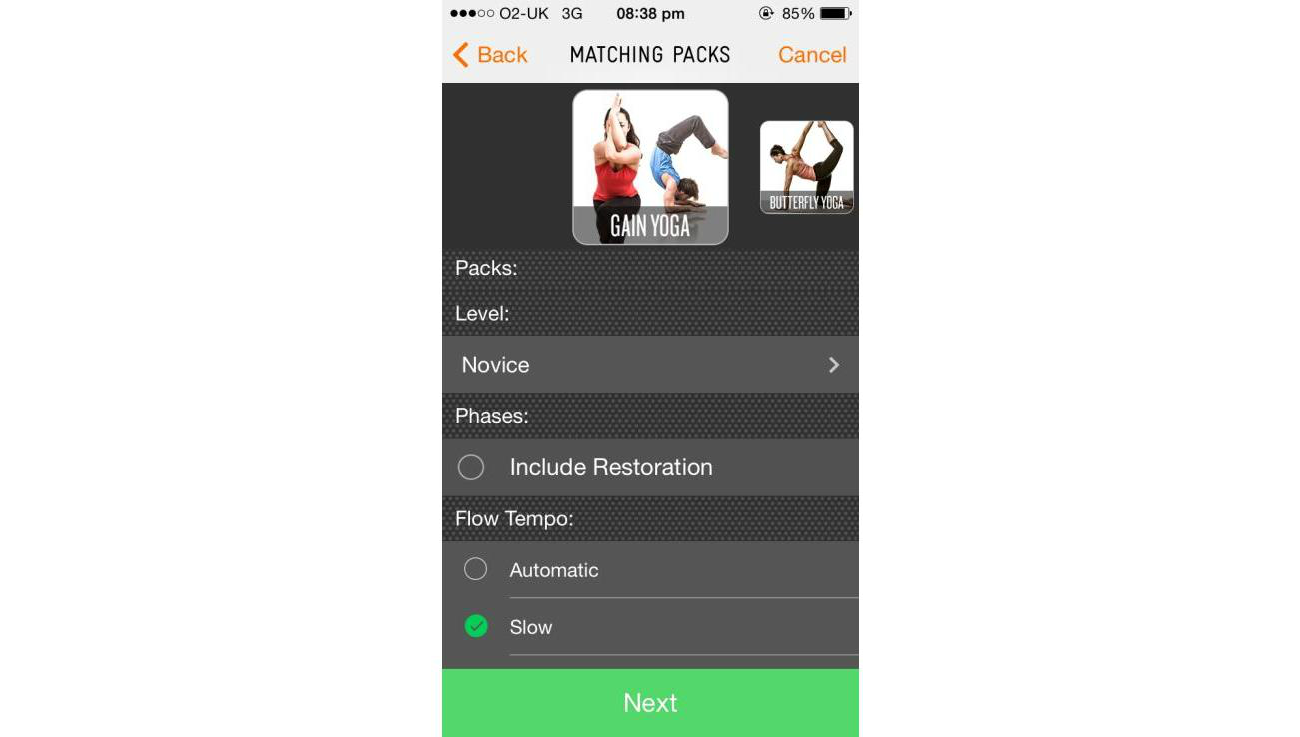
Identify preferences for fitness pack, fitness level, warm up and cool down preferences, and breaks between exercises.

**Figure 4:**
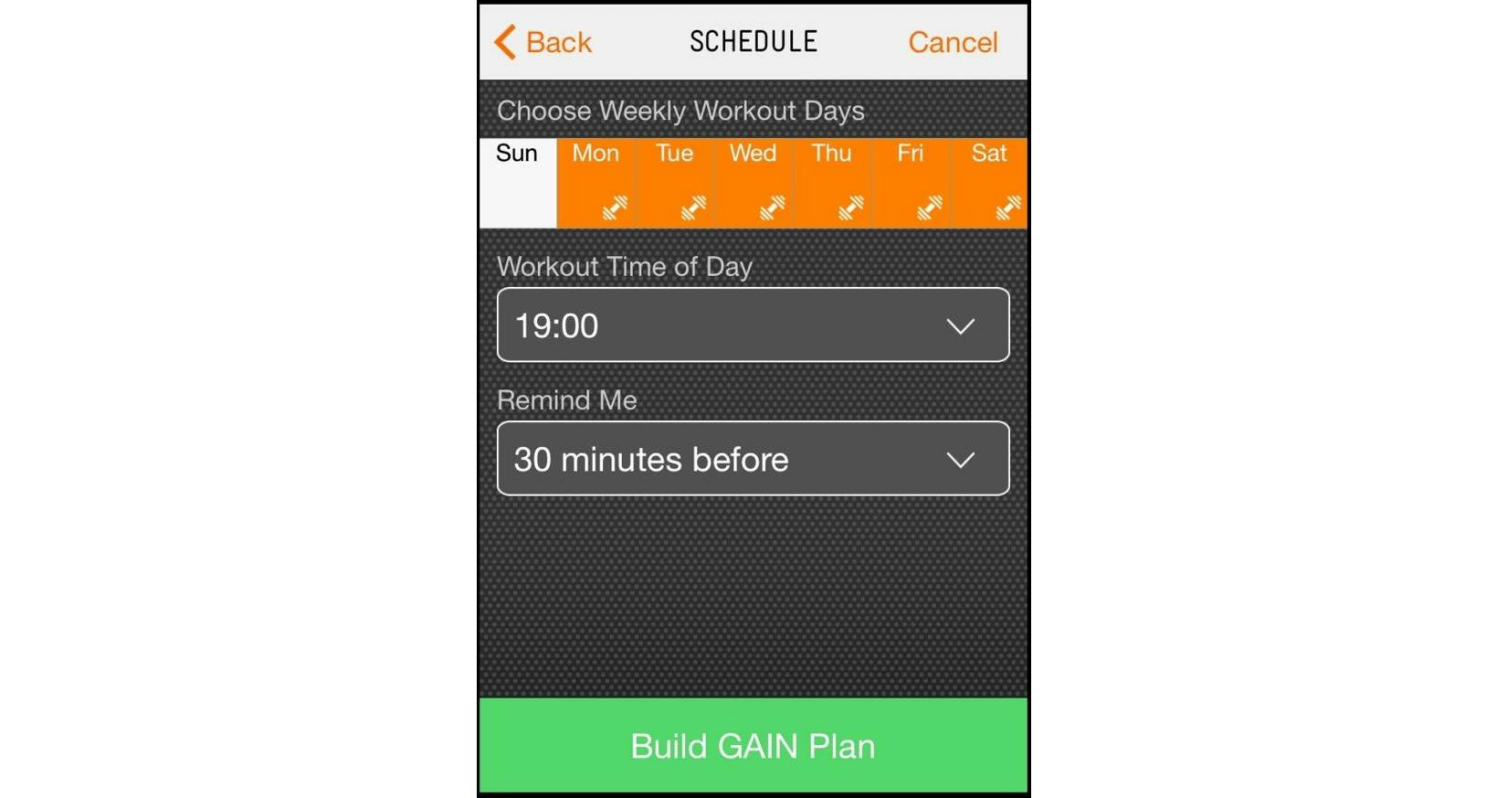
Identify workout schedule and reminders.

**Figure 5:**
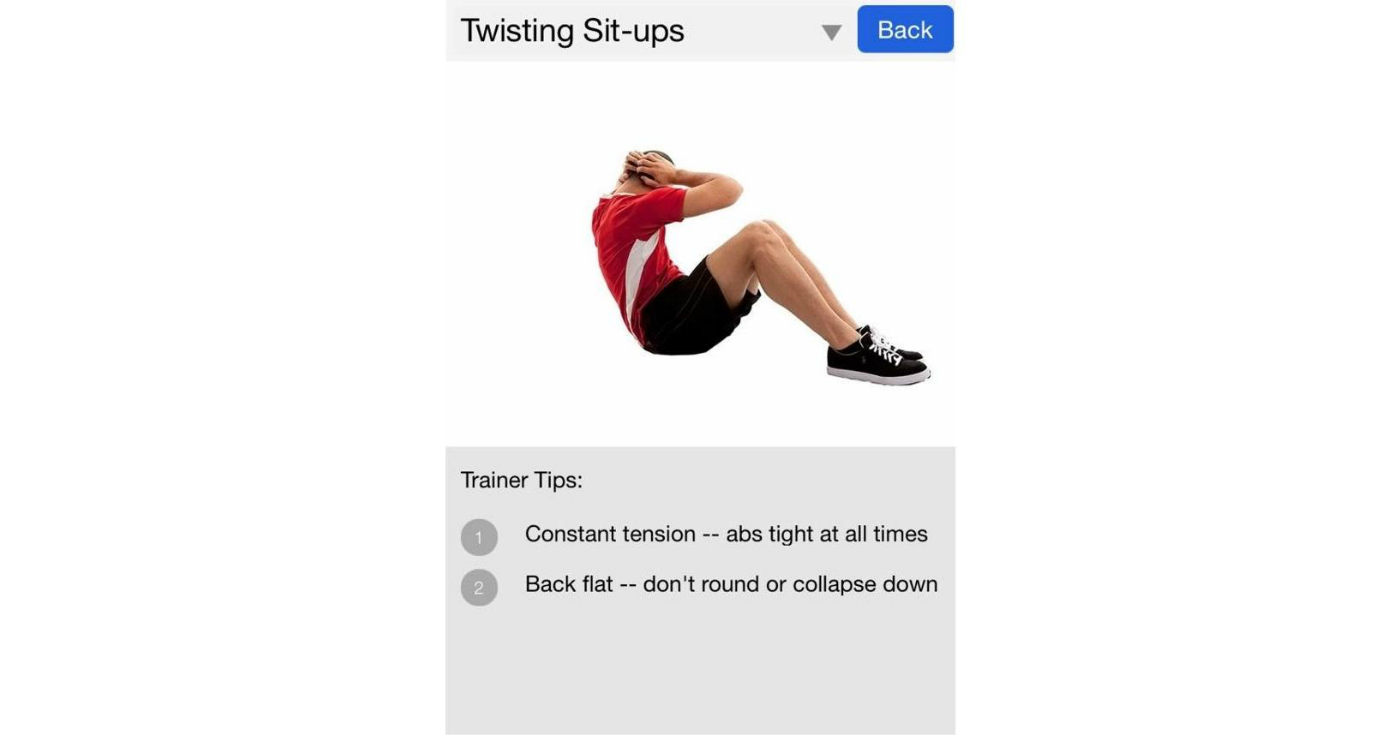
Still image from motion video demonstration and trainer tips instructions on how to perform each exercise.

The app chosen for this study was GAINFitness; a free, self-guided PA app aimed towards the general population. GAINFitness is currently available for download on the iOS operating platform via the Apple App Store. The authors have no association with the developers of GAINFitness. GAINFitness was selected as it provides a PA program based on the user’s goals, current fitness level, and equipment they have access to. Moreover, the app incorporates many features that are common among popular PA apps available for public use. On the first use of the app, users are asked a series of questions to tailor the recommended PA program. First, users are asked to identify their fitness goal, the usual location of their PA (eg, at home, the gym, or on-the-go), their desired duration of a workout, and whether they prefer a balanced workout or wish to focus on a particular muscle group (Figure 1). Users are then asked to identify the pieces of equipment they have access to (Figure 2) so that exercises using appropriate equipment are recommended. The app also tailors the program according to the user’s PA preferences (Figure 3). They are asked to select a free “fitness pack” on the type of PA to perform (cardiovascular fitness, strength training, yoga, pilates). There are other packs available for purchase via the app, which participants could download if they chose to. The PA program is then tailored to the user’s fitness level. The user is required to identify the level of difficulty for their exercises, the “‘flow tempo” so they can increase the length of time for breaks between each exercise, and whether they would like to include a “warm-up” and “cool-down”. The tailoring of the recommended PA program distinguishes GAINFitness from many other PA apps available on the App Store as it is appropriate for users with low baseline PA levels as well as those who are regularly active. Finally, users are asked to identify the days of the week and time of day for their workouts to be completed, and to set a workout reminder (Figure 4). This information is incorporated to set users a 4-week plan tailored to their current PA level, ability, and preferences. After the 4 weeks, the goal can be continued or amended. The tailored workout routines are comprised of individual exercises which participants viewed via video demonstrations, written “Trainer Tips” and spoken instructions on how to perform the exercises correctly (Figure 5). Push notifications were also delivered to the user’s device when a scheduled workout was missed.

To the best of our knowledge, no other study has used GAINFitness as a PA intervention nor is there any published literature regarding its development or theoretical basis. Attempts to contact the developers regarding its development and theoretical underpinnings were unsuccessful. However, the Behavior Change Taxonomy [57] was used to identify behavior change techniques (BCTs) incorporated within the app. BCTs included goal setting (behavior), action planning, review behavior goals, feedback on behavior, self-monitoring of behavior, instruction on how to perform behavior, demonstration of the behavior, graded tasks, prompts and cues, and social reward.

### Qualitative Interviews

Semi-structured interviews were conducted via telephone after the 6-week study period. The interviews aimed to gain in-depth data about the participants’ opinions and experiences having used the app, whether they felt it was appropriate for cancer survivors, and if appropriate, how it could be better adapted to suit their needs. Interviews were recorded with permission of participants, guided by a semi-structured interview schedule (Textbox 1), and were transcribed verbatim. The interviews lasted between 15 to 25 minutes. Two conducted in Polish were translated into English.

**Qualitative semi-structured interview topics.**

Broad interview topic and discussion points

App features

- Which features of GAINFitness did participants like and dislike
- Features that could be improved and/or were missing
- Thoughts about specific features (eg, video instructions)

Types of workout

- Which workout packs they chose to use
- Their opinions of the types of packs which were available
- Which packs were most suited for them in context of cancer

Workout plan

- Did they use the tailored workout program
- Their experience and opinions of using this (or not)

Tailoring to cancer survivors’ needs

- How appropriate the app/exercise program was in promoting PA to cancer survivors
- How could and/or should an app motivate cancer survivors to increase PA (if at all)

Technical experience

- How easy to use was the app
- Any technical issues experienced
- Thoughts about app settings and using the app on a mobile phone from a technical perspective (eg, battery life)

Physical activity

- Thoughts about whether participants felt using app increased their PA
- Stability of those changes (if changes were made)

Pain and injuries

- Did participants experience any pain, injuries or discomfort while exercising using the app

Visuals and layout

- Participants’ opinions of the visual/aesthetic aspect of the app (e.g. layout, color scheme)

Outcomes

- Thoughts about whether participants noticed any changes in physical activity, quality of sleep, fatigue, mood, well-being
- Stability of those changes (if any)

Lifestyle

- How did using the app fit in with their lifestyle and needs in the context of cancer

General comments

- Overall opinions of the app
- Whether they’re intending to continue using the app
- Any other comments

### Outcome Measures

#### Socio-Demographic and Cancer Outcomes

In the baseline survey, participants were asked to report standard demographics including age, gender, education level and ethnicity, and weight and height to calculate body mass index (BMI). The questionnaire also included questions on cancer type, cancer stage at diagnosis, type of treatment(s), and time since primary treatment ended (in months).

#### Engagement With the Mobile App

As the authors have no connection with the developers of GAINFitness, we were unable to obtain objective app usage data (eg, frequency and duration). Instead, participants were asked to complete a log sheet to assess engagement with and usage of the mobile app during the 6-week period. Each time participants used the app they reported the date, duration, and type of each workout (eg, yoga, strength training for legs). Participants reported this information to a research assistant each week via telephone.

#### Physical Activity and Psychosocial Outcomes

Participants completed measures to assess PA and psychosocial outcomes at baseline (0 weeks; T0) and at 6-week follow-up (T1). All measures are valid, reliable, and have been used in previous studies with cancer survivors [44,47,58-60]. PA was assessed using an adapted version of the Godin Leisure-Time Exercise Questionnaire (GLTEQ), which asks participants to report the frequency of performing “strenuous”, “moderate” and “mild” physical activities in the previous week [61]. Strenuous PA includes vigorous activities during which the heart beats rapidly, such as running or long-distance cycling. Moderate PA is considered to be not exhausting and includes activities such as fast walking or easy swimming, whereas mild PA is considered to be of minimal effort and include activities such as yoga or easy walking. The GLTEQ has been previously used in oncology settings [62]. The GLTEQ does not ask about the duration of an exercise session thus a question was added to allow for the calculation of the total number of minutes per week in each type of activity (as recommended by Livingstone [63]). Cancer-related fatigue was assessed using the Functional Assessment of Chronic Illness Therapy (FACIT)-Fatigue Scale Questionnaire [64]. QOL was assessed using the Health and Quality of Life Outcomes (EQ5D) Questionnaire [65], and well-being was assessed using the Functional Assessment of Cancer Therapy-General (FACT-G) Questionnaire [66]. Participants’ sleep quality was analyzed using the Pittsburgh Sleep Quality Index (PSQI), with scores greater than 5 indicating poor sleep quality [67]. Participants’ anxiety and depression scores were assessed using the Hospital Anxiety and Depression Scale (HADS) [68].

### Procedure

On response to study advertisements, participants were contacted to obtain informed consent and were provided with a participant identification (ID) number to enter for the online surveys. Study participants were emailed a URL link to the T0 survey and 6 weeks later to the T1 survey. Both surveys had a “welcome” and “thank you” page. The T0 survey collected information on standard demographics, weight and height, PA, and psychosocial outcomes whereas the T1 survey collected information on PA, weight and height, and psychosocial outcomes only. All questions were mandatory and respondents were able to review and change their answers prior to submission if required. Internet Protocol (IP) addresses and participant ID numbers were used to check for duplicate surveys. Participants completed the log sheets each time they used GAINFitness. After the 6-week period of using the app, participants were telephoned to take part in the qualitative interview (T1). Ethical approval for the study was obtained from University College London Ethics Committee (Reference: 6510/00).

### Analyses

Recruitment success and engagement with the study was evaluated by the number of participants who agreed to participate and completed the study. Self-reported frequency and duration of app use was calculated from log sheets. Changes in T0 and T1 PA and psychosocial outcomes were analyzed using non-parametric tests. Results were considered significant at an alpha level of .05. Qualitative interview data were transcribed verbatim and analyzed using thematic analysis. Initial line-by-line codes were generated and secondary coding involved identifying links between codes to allow for creation of “themes”. Themes were reviewed and defined as coding progressed, and patterns within the data reported.

## Results

### Recruitment and Study Uptake

**Figure 6:**
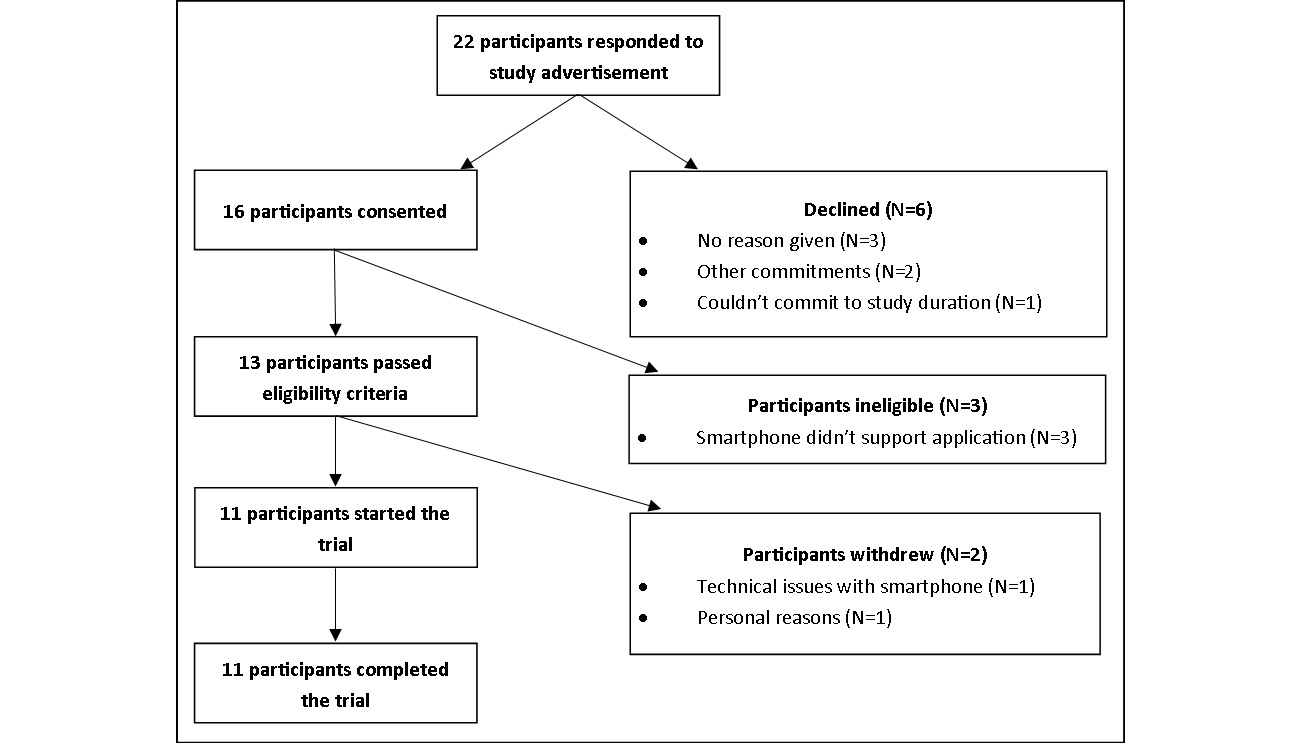
Flow diagram of participants through the study.

The flow of participants through the study is shown in Figure 6. Of the cancer survivors that responded to the study advertisement (N=22), 6 (27%, 6/22) declined participation (Figure 6). The remaining 16 survivors were screened for eligibility. Of those, 3 (19%, 3/16) did not meet eligibility criteria as their mobile phone did not support the app. A total of 13 participants entered the study, and 1 withdrew before baseline measures were taken due to technical issues with their mobile phone and another withdrew for personal reasons. All 11 remaining participants who completed baseline measures completed the trial (100%, 11/11).

### Participant Characteristics

Of the 11 participants who took part in the study, 7 (64%, 7/11) were breast cancer survivors, 2 (18%, 2/11) were colorectal cancer survivors, and 2 (18%, 2/11) were prostate cancer survivors. Participants were mostly female (82%, 9/11), and white British (82%, 9/11). Participants’ age ranged from 33 to 62 years with a mean (SD) age of 45 (9.4). The majority of participants (73%, 8/11) had a BMI in the “normal” range (19-24 kg/m^2^); however one participant was underweight (BMI 17.5 kg/m^2^), and two were overweight (BMI>25 kg/m^2^) (Table 1). At baseline, participants spent a mean (SD) of 72.73 (89.90) minutes in strenuous PA per week, 224.55 (233.38) minutes in moderate PA, and 163.64 (102.50) minutes in mild PA. Participants’ mean (SD) PSQI score at baseline was 9.27 (6.72) indicating that, on average, participants suffered from poor sleep quality [69]. Participants’ mean fatigue scores of 36.20 (11.82) were below the reference score of 40.10 (10.40) for the general population, suggesting they suffered from cancer-related fatigue. The majority of participants (82%,9/11) met the recommended weekly guideline of PA (minimum 150 minutes per week of moderate and strenuous PA).

**Table 1:** Baseline characteristics of study participants (N=11).

Characteristic		n (%)
Gender		
	Female	9 (82)
	Male	2 (18)
Ethnicity		
	White British	9 (82)
	Polish	2 (18)
Body mass index (BMI; kg/m^2^)				
	Healthy	8 (73)
	Underweight	1 (9)
	Overweight	2 (18)
Education level		
	Below degree level	5 (45)
	Undergraduate degree	3 (27)
	Postgraduate degree	3 (27)
Cancer type		
	Breast	7 (64)
	Colorectal	2 (18)
	Prostate	2 (18)
Cancer stage at diagnosis		
	Stage 2	6 (55)
	Stage 3	4 (36)
	Stage 4	1 (9)
Treatments undergone^a^		
	Chemotherapy	8 (73)
	Radiotherapy	7 (64)
	Medication	5 (45)
	Surgery	7 (64)
	Hormonal treatment	2 (18)
	High intensity focused ultrasound	1 (9)

^a^Percentages do not equal 100% as most participants experienced more than one type of treatment.

### Qualitative Interview Findings

All participants completed telephone interviews at the 6-week follow-up to discuss their experiences of using the app during the study period. During these interviews, participants reported that they did not experience any injuries or pain during or following the use of the app. Thematic analyses identified the following four themes: (1) barriers to PA, (2) receiving advice about PA from reliable sources, (3) tailoring the application to one’s lifestyle, and (4) receiving social support from other cancer survivors.

#### Barriers to Physical Activity

The participants discussed some of the barriers they faced towards frequent PA participation and the majority highlighted cancer-related fatigue as the main barrier:

My fatigue is much better now since I finished treatment, but it still gets me bad sometimes.

P4

The only thing that holds me back from exercising frequently, is the fatigue, it’s always the fatigue. So […] if an app somehow could consider my fatigue on those bad days. [Because] it really demotivates you…like you know when you just can’t complete a workout because of it.

P6

Some participants suggested ways in which they felt a PA app could be adapted or developed specifically for cancer survivors and how this could help to overcome fatigue to encourage PA participation:

The app should ask about your fatigue levels […]. When the fatigue is bad, [it] could give you some type of a yoga workout, where you just breathe and stretch and relax. I think that would nice because then you still move, you still do something.

P4

Another said: On days that fatigue is bad you could have a lighter workout, like stretching or walking […], so you still get that recommended 30 minutes [of exercise].

P6

Particularly for those participants who had been diagnosed with breast cancer, lymphedema was also highlighted as a barrier to PA participation due to the fear and confusion surrounding what would be appropriate PA to carry out. In general, the participants felt that the app suggested suitable and safe exercises for dealing with lymphedema and the associated limitations:

I still have problems with my arm [from lymph node dissection surgery], but I didn’t experience any pain with the arm when exercising with the app. Those type of exercises [are] what the doctor tells you to do in the hospital after your surgery anyway.

P9

You can’t put too much pressure on your arms [after lymph node dissection surgery], but you have to train them too to avoid lymphedema. So I think in those terms the application was really good, definitely suitable.

P6

#### Receiving Advice About PA From Reliable Sources

The app featured visual instructions that demonstrated how to perform the exercises correctly. Participants reported that together with the voiceover, the instructions were particularly helpful and made them feel confident about how to perform the exercises correctly:

[The visuals] were really good because [they] showed you how to do everything and you felt confident that you are doing it right.’

P2

Another said: “The visuals were set to be really slow, so I had time to get into position and knew what to do, so I wasn’t worried about any injuries.

P9

I personally am scared of getting lymphedema, and still don’t know sometimes what exercises are good to prevent it, so I think that maybe educating people about […] consequences of not exercising from a really good NHS source would be helpful.

P10

#### Tailoring the App to One’s Lifestyle

The participants reported that the app was suitable for use by cancer survivors and did not cause any injuries or specific problems. However, they believed it could be tailored to better suit the individual’s lifestyle and fitness needs of those who have had cancer:

Anyone with any condition could use this program, which is beneficial, but it could be more beneficial […] more tailored to the type of cancer or disease you had, to your lifestyle and fitness goals. I think it could be more fine-tuned to your circumstances, lifestyle, then that would be really helpful.

P1

I think it should be more of a life context rather than just a general program, so there should be a little bit about what you should do post-treatment, and also in a longer term. At first it should be more about trying to get you more active, […], but once your cancer improves, what are you going to do for the rest of your life? Because you need that fitness to prevent it from coming back.

P1

Several participants highlighted that differentiating between the types of treatment they’d undergone, the types of cancer they’d been diagnosed with, and the associated side effects should be considered when adapting a PA mobile app to cancer survivors’ needs:

The issues I might have as a colorectal cancer survivor are very different from the ones than someone who had breast cancer or prostate cancer.

P8

It is important to think about treatment someone had – I think that different treatments for different cancers have different side effects, and that’s important to consider, because it’s the side effects that stop you from exercising.

P3

It could be fine-tuned better to some of the challenges that I’ve got, like muscle wastage [prostate cancer] and so on, and give me something slightly different to do.

P1

The participants also highlighted the importance of a PA app fitting in with the context of the rest of their life, and in relation to cancer survivorship and health promotion:

It could be even linked through NHS so you could have access to your entire medical stuff and give you a nice history of your progress. If you see that your blood pressure lowered because of exercise, [then] that would motivate you to be more active.

P11

You are told to do 30 minutes of exercise a day, so [getting] something like a reminder telling you that you have completed your half an hour, or how much you have got left [of it] would be really good.

P7

I think the app should maybe have like some health tips you know, like facts about cancer and best ways to be active after treatment.

P4

Several participants also discussed the possibility of an overall cancer survivorship app, rather than just focusing on PA:

I think that the app should help with other things than just exercise; it should be more a lifestyle advice too. [Such as] giving you advice on counselling or tips on how to get better sleep or [listing] foods to eat that could give you more energy.

P3

I think that if an app would ask you about your levels of fatigue and about how you sleep, [it] will be nice, because it will be like some sort of a diary where you can look back at your progress not only in terms of exercise but also your well-being and mood. Something to look back at that documents your recovery, because it will motivate you to keep getting better.

P8

#### Social Support From Other Cancer Survivors

Participants reported that having a social component (eg, forum, social network or ability to add friends) within the app was important to them and this was highlighted as something which was lacking in the current app:

It is so important to get in touch with people who went through the same thing as you have. […] I think that if an app for cancer survivors had a forum on it as a part of the application to motivate each other, that would be amazing.

P11

If you are looking at the issues of cancer survivorship, I think personally that for cancer survivors it would be quite nice to link up with other people and build that community.

P8

Another said:

Also social support of course, that’s good, I use those forums and they are very helpful, even with general stuff, not just exercise. Having support from other cancer survivors is very important.

P5

You do need that bit of motivation from other people. It’s all about motivation when it comes to exercise […]. When you feel low and can’t be bothered to go for a walk, maybe someone else saying‘go on, get up and do it, you can do it’would motivate you.

P2

It was also highlighted that a social support group within the app would be very convenient and a desired component if an app were to be developed and adapted:

We have those support groups in the hospital, but me myself I can’t always make it, because we live far from the hospital. I just got an invitation for one of those and I won’t be attending because I am just too busy. And having that support within an app, without having to leave the house would be really nice, to kind of make some contacts and chat to other people about your experiences.

P9

### Engagement With the Mobile App

Only one participant needed additional help to install the application. All participants kept a record of their app usage throughout the 6 weeks, however, 9 participants used the log sheets provided and 2 chose to use their own means of logging their usage (eg, personal diaries). All 11 participants provided this data during weekly telephone calls with a researcher. Participants used the app a mean (SD) of 2.07 (0.68) times per week, with a mean session duration of 25.08 (8.22) minutes. App use duration ranged from 24.50 to 91.00 minutes per week with a mean (SD) of 44.00 (20.50) minutes. In the qualitative telephone interviews at T1, five participants (45%, 5/11) reported that they would continue using the application, and 100% (11/11) of participants said that they would continue using the app if it was adapted to better suit the needs of cancer survivors.

### Physical Activity and Psychosocial Outcomes

The results from quantitative analyses are shown in Table 2. All 11 participants completed all items in each questionnaire at T0 and T1 so there were no missing data. Ten participants completed the questionnaires on a computer, and 1 participant used a tablet. Wilcoxon signed rank tests showed a significant reduction in reported sleep problems (PSQI) between T0 (median=8, IQR=15) and T1 (Median=6, IQR=10), (*z*=-2.53, *P*=.008). There was a significant increase in participants’ strenuous PA between T0 (median=40, IQR=105) and T1 (median=120, IQR=150), (*z*=-2.80, *P*=.002). There was a signification reduction in participants’ mild PA between T0 (median=150, IQR=90) and T1 (median=80, IQR=120), (*z*=-2.21, *P*=.031).There were no significant changes in other psychosocial outcomes or BMI (Table 2).

**Table 2:** Comparisons of baseline (T0) and the 6-week follow-up (T1) physical activity and psychosocial outcome measures using Wilcoxon signed rank tests.

Outcome		T0, median (IQR)	T1, median (IQR)	*z*	*P* ^a^
Sleep quality^b^ (PSQI)		8.0 (15.0)	6.0 (10.0)	-2.53	.008
Strenuous physical activity, min/week		40.0 (105.0)	120.0 (150.0)	-2.80	.002
Moderate physical activity, min/week		180.0 (150.0)	180.0 (330.0)	-0.76	.563
Mild physical activity, min/week		150.0 (90.0)	80.0 (120.0)	-2.21	.031
Fatigue (FACIT-Fatigue scale)		34.0 (18)	39.0 (14.0)	-1.27	.242
Quality of Life (EQ5D)							
	Mobility	1.0 (1.0)	1.0 (0.0)	-1.41	.500
	Self-care	1.0 (0.0)	1.0 (0.0)	0.00	1.000
	Activity	1.0 (1.0)	1.0 (1.0)	0.00	1.000
	Pain	2.0 (1.0)	2.0 (1.0)	-1.00	1.000
	Anxiety	1.0 (1.0)	1.0 (1.0)	-1.00	1.000
Well-being (FACT-G)		40.0 (7.0)	47.0 (10.0)	-1.85	.064
Anxiety (HADS-anxiety scale)		4.0 (8.0)	3.0 (7.0)	-1.61	.137
Depression (HADS-depression scale)		2.0 (5.0)	2.0 (6.0)	-0.32	.844
BMI		23.9 (5.2)	23.4 (5.0)	-0.25	.828

^a^Exact significance.

^b^Higher PSQI scores indicate increased reported sleeping problems.

## Discussion

### Principal Findings

The present study utilized a one-arm, pre-post, mixed-methods design to examine experiences of using a publicly available PA mobile application (GAINFitness) in breast, prostate, and colorectal cancer survivors. All participants (N=11) engaged with the app and qualitative interviews highlighted that the app was well-received. Recommendations were identified on how a PA app may be adapted or developed to increase the relevance and suitability for cancer survivors. Willingness and ability to complete the quantitative PA and psychosocial measures was established as all participants completed survey measures at both time points, with no missing data or reports of dissatisfaction with measurements or study procedures. Significant increases in strenuous PA participation, improvements in sleep quality, and reductions in mild PA participation were observed. There were no significant changes for any other PA or psychosocial outcomes.

Qualitative telephone interviews investigated cancer survivors’ attitudes towards GAINFitness in order to understand the appropriateness of the app for use in this population. Findings from this study suggest that the app and this approach to intervention delivery were well received. Given the rising use of mobile phones and mobile technology [48], and the popularity of mHealth [49], this approach to intervention delivery is timely. However, important barriers relating to PA in the context of cancer were highlighted. GAINFitness did not address these barriers and PA apps should be adapted to overcome such barriers and thus improve suitability for cancer survivors.

Interviews showed that video demonstrations twinned with voiceover instructions explaining how to do exercises were valued by participants. The videos provided participants with reassurance that they were performing the exercises correctly and safely. This, in combination with their desire to receive PA recommendations and advice following a cancer diagnosis from reliable (eg, NHS) sources highlights a lack of knowledge of PA and a lack of confidence to perform PA among cancer survivors. Similar findings have been previously reported. For example, one study showed that cancer survivors feel they are given insufficient information regarding PA, diet, and weight [70]. Other studies have shown that health professionals demonstrated inadequate awareness and low information provision of PA and lifestyle guidelines specifically for cancer survivors [71,72]. Lack of time during consultations has been found to be a barrier to discussing PA with cancer survivors by health professionals [71]. Further, a Macmillan Cancer Support report highlighted that over half of health professionals know little or nothing about the benefits of PA in prevention or management of the side-effects and long-term outcomes of cancer [73]. The same report found that only 6% of health professionals discuss PA with patients with cancer. In the present study, breast cancer survivors highlighted their concerns about PA in relation to lymphedema which may be experienced after breast surgery and treatment. They discussed confusion surrounding prevention and risk of developing lymphedema in relation to PA participation and were unaware of appropriate exercises to reduce risk. Similar findings are reported by Sander and colleagues [74]. Taken together, this research illustrates the need for better awareness and understanding of the evidence for the benefits of PA for cancer survivors among health professionals and better information provision for patients. An evidence-based PA app developed specifically for cancer survivors could be recommended by health professionals. Such an app may provide PA guidance and reassurance surrounding exercises that are safe and appropriate for cancer survivors. Moreover, simply recommending an app would have minimal impact on the time constraints of consultations. Participants in the current study suggested that a PA app for cancer survivors should incorporate a feature to recommend appropriate exercises for specific cancer types.

In interviews, cancer-related fatigue was consistently discussed as an important barrier to PA participation. This supports previous literature that has found similar findings [4,41,42]. It is plausible to assume that fatigue is an important factor in the low proportion of cancer survivors meeting PA guidelines [38,39] and the observed fall in PA participation following a cancer diagnosis [40]. It is likely that cancer survivors and health professionals are unaware of the benefits of PA participation in relation to cancer-related fatigue. Given this evidence and the feedback from participants in the current study, it is necessary for a PA intervention for cancer survivors to include gradual increases in PA participation, with the option to begin with lower intensity PA and greater education surrounding the benefits of PA in combating cancer-related fatigue. The participants in the current study also suggested that a PA app tailored specifically for cancer survivors could ask users to report their level of fatigue and recommend a lower intensity program when fatigue is particularly high to encourage them to participate in some PA, rather than avoid it altogether.

The participants’ feedback pertaining to the desire for a feature within the app for social support was also highlighted. The participants felt that it was important to build a sense of community among cancer survivors and an environment in which they could share their experiences and support each other to increase PA participation. This supports findings from a similar Web-based PA intervention for older cancer survivors in which the social community within the program was particularly well-received [54,55]. It is therefore recommended that a PA app specifically aimed towards cancer survivors should incorporate this as an intervention component.

The quantitative outcome measures and online approach to data collection was intended to model the process and outcome of intervention evaluation in line with MRC guidance [56]. It was intended that this could provide an indication of the potential for change in PA and psychosocial outcomes, rather than as a reliable evaluation of the efficacy of the app, which could be tested in a future RCT. It is encouraging that significant increases in strenuous PA participation and improvements in sleep quality were observed during the 6-week period. There were significant decreases in mild PA participation. One plausible explanation is that participants displaced mild PA with vigorous. There were no other significant changes in moderate PA participation or other psychosocial outcome measures. It is possible that a more targeted PA app may demonstrate greater improvement, which could be more reliably investigated using an RCT.

The current study should be viewed in light of a number of limitations. The majority of participants were white, female, breast cancer survivors. Therefore, the results may not be generalized to other cancer survivor populations. Participants in the present study had high baseline levels of PA. Owing to limited awareness of the benefits of PA and guidelines for cancer survivors among health professionals [71,73], it is plausible that many patients are equally unaware and so only those who are motivated to be physically active volunteered to take part. The recruitment method utilized for the present study meant that calculation of a response rate of the number of participants who agreed to take part as a proportion of the number of eligible people who viewed the advertisements was not possible. It would be necessary to use more targeted recruitment strategies in future studies such as in-clinic approaches, referrals from health professionals or via face-to-face cancer support services and charities. These approaches would also allow for a calculation of the response rate which could help to determine acceptability of the intervention. However, it is reassuring that 86% (19/22) of eligible participants who contacted the study team in response to advertisements were willing to take part in the study, and all 11 participants who started the study completed it. While the GLTEQ has been frequently used among cancer survivors, future studies should aim to use objective measurements of PA (eg, accelerometers) and this would certainly be recommended in a formal evaluation of a PA app. The app selected is also currently only available on the iOS operating system and four participants had to be excluded as their mobile phone did not support the application. Finally, this study was a one-arm pre-post design study, without a control group and so we cannot be sure that the significant changes are as a result of the app. However, for the quantitative part to this study, the aims focused on modeling the data collection process and outcomes and assessing the likelihood for behavior change. The qualitative feedback surrounding the usability and content of the app can now be taken forward for intervention development, which can then be evaluated in a formal RCT.

### Conclusion

The current study demonstrates that cancer survivors engaged with a PA app and this approach to intervention delivery was well-received. However, important factors which are not included in GAINFitness were highlighted. This included not accounting for the effects of cancer-related fatigue, the lack of information provision surrounding PA participation in the context of cancer from reliable sources, the need to consider limitations associated with specific cancer types in relation to PA, and the desire for a way to receive social support from other cancer survivors within the app. There is potential for change in PA and psychosocial outcomes among cancer survivors through the use of this publicly available PA app, however, we recommend that a more targeted PA app aimed towards cancer survivors may increase the relevance and suitability of the intervention for this population and may prove more effective. The findings of this study can be taken forward for intervention development to adapt or develop a PA app for cancer survivors, which should be tested in a larger RCT with objective measures of PA.

## Abbreviations

BCT: behavior change technique
BMI: body mass index
EQ5D: Health and Quality of Life Outcomes Questionnaire
FACIT-Fatigue: Functional Assessment of Chronic Illness Therapy -Fatigue Scale Questionnaire
FACT-G: Functional Assessment of Cancer Therapy-General,
GLTEQ: Godin Leisure Time Exercise Questionnaire
HADS: Hospital Anxiety and Depression Scale
ID: identification
LWBC: Living With and Beyond Cancer
mHealth: mobile health
MRC: Medical Research Council
NHS: National Health Service
PA: physical activity
PSQI: Pittsburgh Sleep Quality Index
QOL: quality of life
RCT: randomized controlled trial
TO: time at baseline
T1: time at the 6-week follow-up
